# Disrupted functional network connectivity predicts cognitive impairment after acute mild traumatic brain injury

**DOI:** 10.1111/cns.13430

**Published:** 2020-06-25

**Authors:** Fengfang Li, Liyan Lu, Song'an Shang, Lanyue Hu, Huiyou Chen, Peng Wang, Hong Zhang, Yu‐Chen Chen, Xindao Yin

**Affiliations:** ^1^ Department of Radiology Nanjing First Hospital Nanjing Medical University Nanjing China; ^2^ Department of Radiology The Affiliated Jiangning Hospital of Nanjing Medical University Nanjing China

**Keywords:** cognitive impairment, functional network connectivity, mild traumatic brain injury, resting‐state fMRI

## Abstract

**Aims:**

This study aimed to detect alterations of brain functional connectivity (FC) in acute mild traumatic brain injury (mTBI) and to estimate the extent to which these FC differences predicted the characteristics of posttraumatic cognitive impairment.

**Methods:**

Resting‐state fMRI data were acquired from acute mTBI patients (n = 50) and healthy controls (HCs) (n = 43). Resting‐state networks (RSNs) were established based on independent component analysis (ICA), and functional network connectivity (FNC) analysis was performed. Subsequently, we analyzed the correlations between FNC abnormalities and cognitive impairment outcomes.

**Results:**

Altered FC within the salience network (SN), sensorimotor network (SMN), default mode network (DMN), executive control network (ECN), visual network (VN), and cerebellum network (CN) was found in the mTBI group relative to the HC group. Moreover, different patterns of altered network interactions were found between the mTBI patients and HCs, including the SN‐CN, VN‐SMN, and ECN‐DMN connections. Correlations between functional disconnection and cognitive impairment measurements in acute mTBI patients were also found.

**Conclusion:**

This study indicated that widespread FNC impairment and altered integration existed in mTBI patients at acute stage, suggesting that FNC disruption as a biomarker may be applied for the early diagnosis and prediction of cognitive impairment in mTBI.

## Graphical abstract

Widespread functional network connectivity (FNC) impairment and altered integration existed in acute mTBI, suggesting that FNC disruption as a biomarker may be applied for the early diagnosis and prediction of cognitive impairment in mTBI.

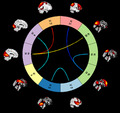

## 1 INTRODUCTION

Mild traumatic brain injury (mTBI) patients have early cognitive impairment, which is mainly related to attention, executive, memory, and language deficits.^1^ It is estimated that up to 40%‐50% of mTBI patients have cognitive impairment symptoms 3 months after injury, and 10%‐25% still have symptoms after 1 year.^2^ Nevertheless, most mTBI patients with cognitive impairment tend to be clinically neglected, given their negative conventional neuroimaging manifestations on computed tomography (CT) and magnetic resonance imaging (MRI).^3^ Therefore, patients with cognitive impairment may be underestimated, resulting in a substantial impact on their life and social interactions. Posttraumatic cognitive impairment might be associated with changes in brain function, which can be observed using resting‐state functional magnetic resonance imaging (rs‐fMRI) that may reveal disorders of large neural networks involved in cognitive function and their relationship with cognitive impairment among mTBI populations.^4^ However, heterogeneity in neuroimaging studies has led to diversity in the systematic understanding of the relationship between cognitive dysfunction and brain states in mTBI patients.

Previous studies have confirmed that changes in functional connectivity (FC) were associated with posttraumatic cognitive impairment in mTBI patients,^5^, ^6^ which mainly focused on the default mode network (DMN),^7^, ^8^ executive control network (ECN),^9^ motor network,^10^ and interhemispheric network connectivity,^11^ suggesting that neuroimaging has potential significance for understanding the neuropathological mechanisms of cognitive impairment after mTBI. Nevertheless, other functional networks related to widespread neurocognitive impairments exist in patients with mTBI, such as the salience, visual, and attention networks, have been less explored.

There has been a growing consensus that posttraumatic cognitive abnormalities reflect atypical interactions between multiple systems in the brain rather than problems affecting isolated brain regions.^12^ Using rs‐fMRI to study interactions between specific brain regions, FC studies have shown that mTBI was associated with abnormal connections in corticocortical networks that support social cognitive functions such as executive function, working memory, and visual attention.^13^ Considering the topological properties of whole‐brain networks, graph theory studies have consistently reported changes in local and global information transmission efficiency in patients with mTBI.^14^ In addition, Li et al^15^ used Granger causality to analyze the topological properties of functional brain networks in mTBI patients based on source connectivity analysis of resting‐state magnetoencephalographic (MEG) activity and identified that the brain regions in which the ingoing and outgoing connections were significantly different across the two groups in different frequency bands. Although these studies help to describe the disconnection model of mTBI, the inter‐network interactions in patients with mTBI have not been comprehensively assessed.

To date, only a few studies have explored inter‐network interactions in mTBI patients, reporting significantly increased connectivity between the CN and SMN^16^ and decreased connectivity between the DMN and basal ganglia network in patients with mTBI.^17^ While these studies have provided initial evidence regarding abnormalities in inter‐network interactions in mTBI, they focused on a limited number of a priori selected networks and did not analyze the interactions between each network. To define different remote interaction patterns, independent component analysis (ICA) has been widely used for identifying resting‐state networks (RSNs) in view of its ability to isolate various brain function networks.^18^, ^19^ Meanwhile, the function network connectivity (FNC) can be used to represent the temporal correlation between these RSNs.^20^, ^21^ Therefore, exploration of the RSNs and FNC may provide more information to advance the understanding of the underlying neural mechanisms of cognitive impairment in mTBI.

Given the basis of prior work and theoretical considerations, we aimed to systematically explore the alterations in RSNs and the interactions between RSNs in acute mTBI. The temporal correlation of brain networks' activity was used to quantify their interactions and to estimate the extent to which group differences could predict the characteristics of posttraumatic cognitive impairment. We hypothesized that distinct FC patterns may exist in acute mTBI patients, and these group differences in RSNs and network interactions would be associated with cognitive impairment.

## 2 MATERIALS AND METHODS

### 2.1 Subjects and clinical data

A total of 53 patients with mTBI (25 males and 28 females; age range: 20‐58 years) were recruited from the emergency department of our hospital between January 2018 and May 2019. The inclusion criteria for patients with mTBI were as follows: (aa) age 20 years or older (right‐handed); (b) the presence of trauma to the head; (c) an initial Glasgow Coma Score (GCS) of 13‐15 in the emergency department; (d) initial emergency room evaluation of mTBI (loss of consciousness <30 minutes, posttraumatic amnesia <24 hours, or recorded alteration in mental status [ie, dazed, confused, or disoriented]); and (e) CT scan as a part of their clinical evaluation. The exclusion criteria were as follows: (a) a history of a previous brain injury, neuropsychological or neurological disorder, psychoactive medication use, or concurrent substance abuse; (b) history of alcohol or drug abuse; (c) history of sedative use in hospitals or emergency rooms; and (d) MRI contraindications. Additionally, 43 healthy subjects (all right‐handed; 19 males and 24 females; age range: 20‐59 years) matched for age, sex, and education level were recruited as the healthy control (HC) group. The HC group was subject to the same exclusion criteria as the patient group. All HCs underwent the same neuroimaging tests as mTBI patients.

### 2.2 Cognitive function assessment

The Montreal Cognitive Assessment (MoCA) was chosen to evaluate the neurocognitive state of all participants, which evaluated several aspects of cognitive function, including visuospatial/execution, naming, language, attention, abstraction, memory (short‐term immediate and deferred recall), and localization.^22^ The assessment time was approximately 10 minutes, with a maximum score of 30. Those subjects with scores of 26 or more were considered cognitively normal, and lower scores indicated poorer cognitive abilities.

### 2.3 MRI acquisition

All MRI data were acquired within 7 days postinjury on a 3.0 Tesla MRI scanner (Ingenia, Philips Medical Systems) using an 8‐channel digital head coil receiver and parallel imaging technology. For this analysis, the functional images were acquired axially using a gradient echo‐planar imaging sequence. The scanning parameters were as follows: echo time (TE) = 30 ms; repetition time (TR) = 2000 ms; slices = 36; gap = 0 mm; thickness = 4 mm; field of view (FOV) = 240 mm × 240 mm; flip angle (FA) = 90°; and acquisition matrix = 64 × 64. The rs‐fMRI sequence scan took 8 minutes and 6 seconds. Structural images were acquired with a three‐dimensional turbo fast echo (3D‐TFE) T1WI sequence with high resolution, and the scanning parameters were as follows: TR/TE = 8.1/3.7 ms; slices = 170; thickness = 1 mm; gap = 0 mm; acquisition matrix = 256 × 256; FA = 8°; and FOV = 256 mm × 256 mm. The structural sequence took 5 minutes and 28 seconds to complete. During all resting‐state scans, scanner noise and head motion were reduced using earplugs and foam padding, and the participants were instructed to close their eyes and rest peacefully. In addition, 3D gradient echo susceptibility‐weighted imaging sequences (TR/TE = 22/34 ms; FA = 20°; matrix = 276×319; slice thickness = 1 mm; and FOV = 220 mm × 220 mm) were also implemented to help detect hemorrhagic or other lesions.

### 2.4 Data preprocessing

Data Processing & Analysis for Resting‐State Brain Imaging (DPABI_V4.3_200301) with the following stages was applied for data analysis.^23^ First, the first 10 volumes were removed from each time series to allow for participant adaptation to the scanning environment. The remaining images were then slice‐timing corrected and calibrated for head motion correction. Participant data demonstrating head movement >2.0 mm translation or >2.0° rotation were excluded from the analysis. The rest of the dataset was spatially normalized to a template from the Montreal Neurological Institute (resampled voxel size = 3×3 × 3 mm^3^), followed by a 6‐mm spatial sequence with a Gaussian smoothing kernel. Finally, several sources of spurious variances were removed by linear regression, which included six head motion parameters, and average signals from white matter, cerebrospinal fluid, and whole brain.

### 2.5 Independent component analysis

The group ICA software of fMRI toolbox software (http://icatb.sourceforge.net/) was used to select RSNs. The ICA analysis was carried out in three stages: (a) data reduction, (b) application of the ICA algorithm, and (c) back reconstruction for each individual subject. The number of independent components (ICs) was determined by using the minimum description length (MDL) criteria.^24^ The data reduction was followed by a set of spatial ICA, which was executed on the aggregate data of the participants, resulting in an estimate of the ICs.^25^ Then, the strength value of connectivity within each independent component was converted into a z‐score to reflect the degree of correlation between the time series of a given voxel and the average time series of its corresponding components. In addition, to verify the choice of separated ICs, we implemented the ICA (model sequence) with different independent component numbers and performed the following analysis (ie, within‐RSN analysis and FNC analysis) seven times.

### 2.6 Within‐RSN analysis

**Figure 1:**
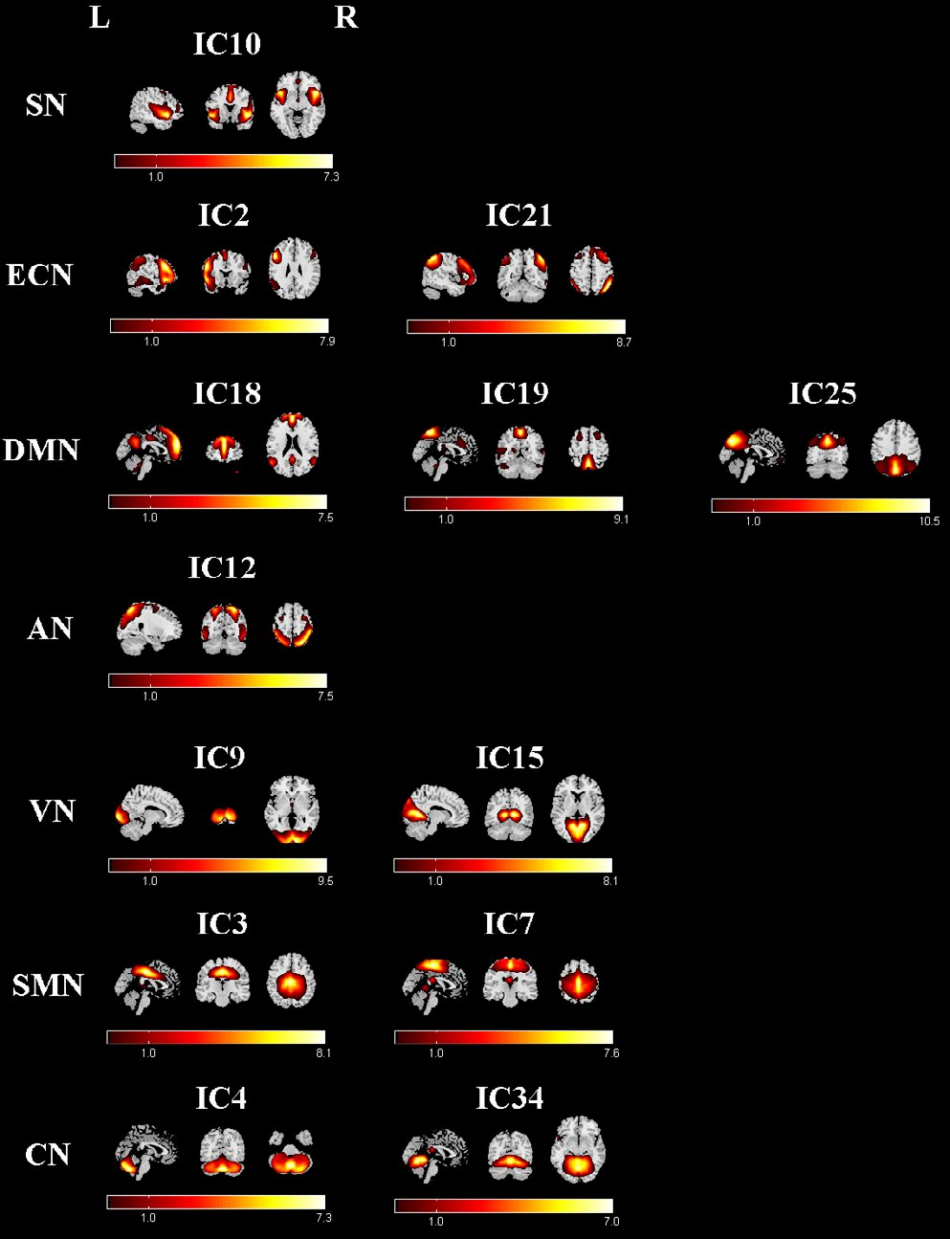
Functional relevant RSNs. The spatial maps of 13 independent components (ICs) were selected as the RSNs for further analysis. AN, attention network; CN, cerebellum network; DMN, default mode network; ECN, executive control network; SMN, sensorimotor network; SN, salience network; VN, visual network

Among the 34 components resulting from ICA, we selected 13 components (7 nonartifactual RSNs) as the focus of the subsequent analyses (Figure 1) through visual inspection in accordance with previous rs‐fMRI studies.^21^, ^26^ For each RSN, the single‐sample *t* test was first used to obtain the z‐maps for each group, the false discovery rate (FDR) was used to correct, and the statistical figure was obtained at the threshold of *P* < .01. Then, the group comparison of the z‐maps of the RSNs was conducted using a two‐sample *t* test restricted to the voxels within a union mask and was determined by the *t* test results of two single samples. Between‐group effects were thresholded at *P* < .01, corrected by FDR correction. Regions with significant differences in the two‐sample t test were chosen from each RSN and used in the subsequent analysis.

### 2.7 Static FNC analysis between RSNs

After the ICA, the individual‐level time courses of recognized RSNs were deduced by using the spatiotemporal double regression method. To study the relationship between time courses of different RSNs, the static FNC analysis was carried out. First, a time‐domain band‐pass filter (band‐pass 0.00‐0.25 Hz) was used to reduce the influence of low‐frequency drift and high‐frequency physiological noise on the time process. The correlations between any two RSN time processes for each participant were then calculated. We calculated FNC using the Pearson correlation coefficient between each and every other summary time course, which resulted in an FNC matrix with the dimensions of 13 times 13 (RSNs) times 93 (participants). In the general linear model, FNC group difference estimation was performed for each pair of RSNs. The significance threshold was *P* < .01, corrected for multiple comparisons using FDR. Then, we focused on RSN pairs with significant differences between the adjusted FNC groups to study their relationship with cognitive performance.

### 2.8 Correlation analysis

The correlations were calculated between FC in the RSNs/FNC and the cognitive function assessments in mTBI. For each RSN, the brain region with a significant difference in the two‐sample t test was selected as the region of interest (ROI) to extract the coordinates of the ROIs. The relevant calculation was then performed using the mean z‐scores within the ROI. In addition, the FNC coefficients that showed a highly significant difference between two groups were also used in the correlations with the MoCA scores.

### 2.9 Statistical analysis

Demographic and clinical characteristic were assessed between mTBI patients and HCs using an independent t test for continuous variables and a chi‐square test for proportions by the SPSS 19.0 software package (SPSS, Inc). Statistical significance was set as *P* < .05. Shapiro‐Wilk tests were used to evaluate data normality, and *P* values >.05 indicate a normal distribution of data. Then, we used Cohen's d to describe the effect size (ES) of each clinical variable. For RSN and FNC analysis, group comparisons between the mTBI and HC groups were performed using two‐sample t tests. The significance threshold was set at *P* < .01 using FDR corrections. Age, sex, and education level were used as covariates. In addition, Pearson's correlation coefficients between functional connection strength and cognitive scores were analyzed with a significance level of *P* < .05. SPM12 was used for voxel‐level statistical analysis of the RSNs, and MATLAB (MATLAB 2013a) was used for other statistical analyses, including FNC group comparisons and correlation analyses.

## 3 RESULTS

### 3.1 Participants and clinical data

After the fMRI data head motion check, three patients were excluded because of excessive head motion artifacts, so the final cohort in this study consisted of 50 mTBI patients and 43 HC subjects. MRI scans were obtained in the mTBI patients at an average of 3.12 days (range, 0‐7 days) after head injury. Compared with the HCs, mTBI patients in the acute stage showed lower MoCA scores (*P* = .000), indicating more severe cognitive disability in the mTBI group. Among all subcategories of the MoCA test, only the visuospatial/executive (*P* = .004), language (*P* = .010), and attention (*P* = .005) subcategories were significantly lower in the mTBI group than those in the HC group. The scores for memory, abstraction, naming, and orientation did not differ between the mTBI and HC groups (*P* > .05). The demographic and neuropsychological data from this study are shown in Table 1.

**Table 1:** Demographic characteristics and cognitive performance in patients with mTBI and healthy controls

Characteristics	mTBI (n = 50)	Controls(n = 43)	*P*‐value	ES
Age (y)	43.82 ± 7.88	41.65 ± 7.87	.189	0.27
Education (y)	12.66 ± 2.96	13.63 ± 3.49	.152	0.29
Sex (Female/ Male)	26/24	24/19	.835	‐
MoCA scores	24.42 ± 2.35	25.65 ± 1.76	.000*	0.59
Visuospatial/executive	3.50 ± 0.90	4.07 ± 0.96	.004*	0.61
Naming	2.78 ± 0.50	2.81 ± 0.39	.722	0.07
Attention	5.36 ± 0.94	5.81 ± 0.45	.005*	0.61
Language	2.40 ± 0.60	2.70 ± 0.46	.010*	0.56
Abstraction	1.84 ± 0.48	1.93 ± 0.25	.325	0.23
Memory	2.78 ± 1.05	3.09 ± 1.26	.197	0.26
Orientation	5.76 ± 0.43	5.91 ± 0.29	.062	0.40

Note

Data are the mean ± standard deviation.

Abbreviations: ES, effect size; GCS, Glasgow Coma Scale; MoCA, Montreal Cognitive Assessment; mTBI, mild traumatic brain injury.

* *P* < .05.

### 3.2 ICA and component selection

In this study, a total of 34 ICs were extracted by ICA, among which 13 components were selected as the RSNs for further analysis in accordance with previously published results (Figure 1). Subsequently, seven networks with these components were labeled as follows: the salience network (SN) (IC10) showed spatial patterns mainly consisting of the dorsal anterior cingulate (dACC) and anterior insular cortices, as well as part of the prefrontal areas. The executive control network (ECN) (IC2 + 21) included the left lateral frontoparietal network (LFPN) and the right lateral frontoparietal network (RFPN). The LFPN was mainly focused at the left middle frontal gyrus, inferior parietal lobule, superior parietal lobule, and angular gyrus; the RFPN mainly showed similar spatial patterns with the LFPN. The default mode network (DMN) (IC18 + 19+25) typically included the posterior cingulate cortex (PCC), precuneus, inferior parietal, bilateral angular gyrus, and medial prefrontal cortex (MPFC) nodes. The attention network (AN) (IC12) mainly included the bilateral intraparietal sulcus, frontal eye field, and middle temporal lobe. The visual network (VN) (IC9 + 15) included the primary visual cortex (the bilateral calcarine sulcus and medial extra‐striate regions [eg, the lingual gyrus and cuneus]) and extravisual (the occipital pole, the lateral occipital cortex, and the occipital part of the fusiform gyrus). The sensorimotor network (SMN) (IC3 + 7) comprised SMN1 that included the supplementary motor area, the paracentral lobule, and the pre‐ and postcentral gyri; SMN2 was mainly focused at the bilateral primary somatosensory cortex, including precentral and postcentral gyri areas. The cerebellum network (CN) (IC4 + 34) had spatial patterns that primarily encompassed the cerebellum anterior lobe, cerebellum posterior lobe, and declive.

### 3.3 Group FC differences within RSNs

**Figure 2:**
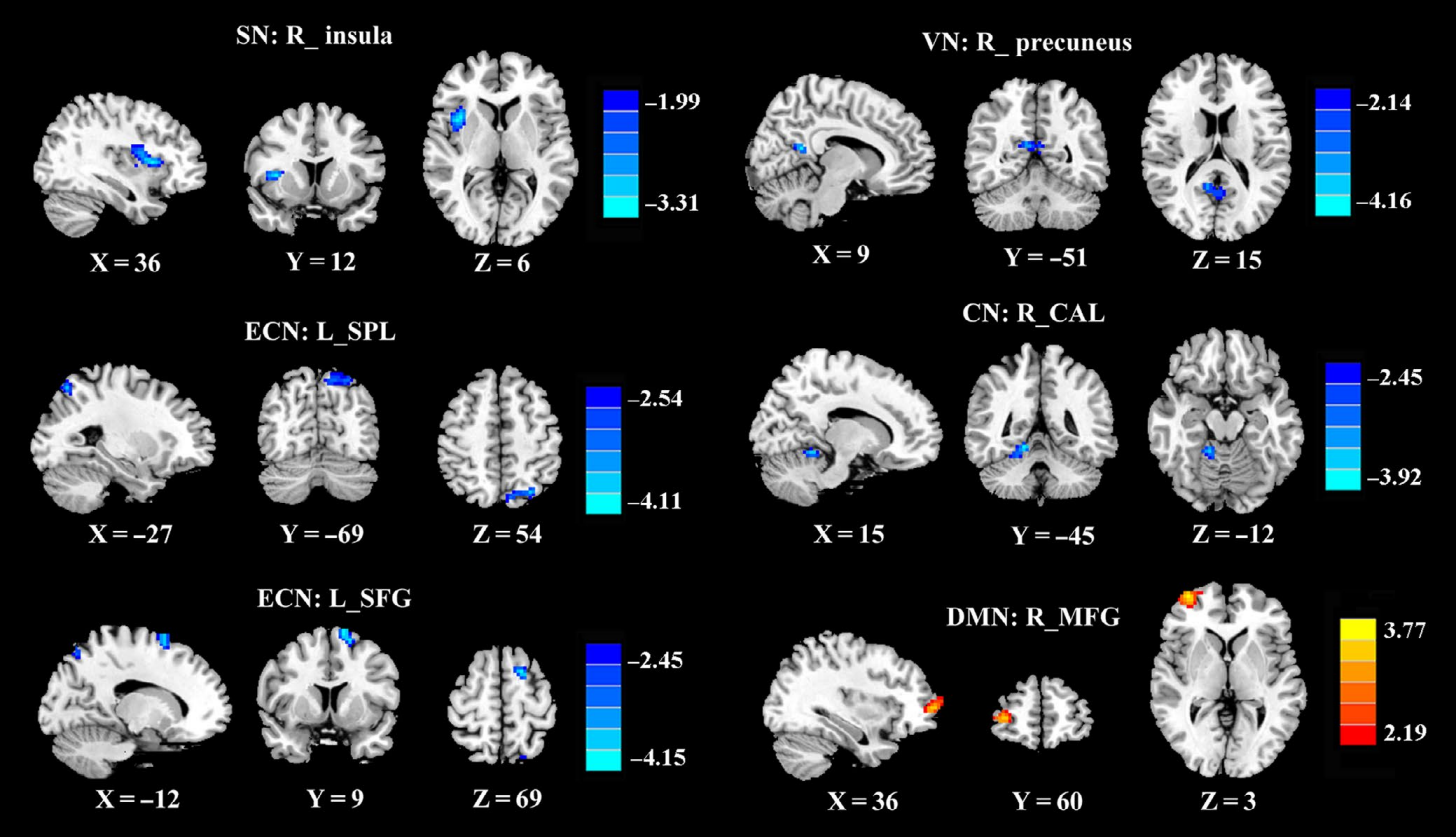
Group FC differences within RSNs. Significant differences between the mTBI and HC groups were found within five RSNs. CAL, cerebellum anterior lobe; CN, cerebellum network; DMN, default mode network; ECN, executive control network; L, left; MFG, middle frontal gyrus; R, right; SFG, superior frontal gyrus; SN, salience network; SPL, superior parietal lobe; VN, visual network

Significant differences between the mTBI and HC groups were found within five RSNs, including the SN, DMN, ECN, VN, and CN (Table 2, Figure 2). Compared with the HC group, the mTBI group exhibited decreased FC within the SN (right insula [R_ insula]), ECN (left superior parietal lobe [L_SPL] and left superior frontal gyrus [L_SFG]), VN (right precuneus [R_precuneus]), and CN (right cerebellum anterior lobe [R_CAL]). Moreover, there was increased FC in the right middle frontal gyrus (R_MFG) in the DMN in the mTBI group compared with the HC group. Based on voxel‐wise analysis, the acute stage mTBI group compared with the HC group did not demonstrate altered resting‐state FC in the SMN and attention network.

**Table 2:** Brain regions with significant differences connectivity within RSNs between acute mTBI patients and healthy controls

	Brain regions	BA	Peak MNI coordinates x, y, z (mm)	Peak *T* value	Voxels
SN	R_ insula	13	36, 12, 6	−3.4331	72
ECN	L_SPL	7	−27, −69, 54	−3.8503	73
	L_SFG	6	−12, 9, 69	−4.4261	74
VN	R_ precuneus	29	9, −51, 15	−4.5638	94
CN	R_CAL	20	15, −45, −12	−4.1842	98
DMN	R_MFG	10	36, 60, 3	3.9161	88

Abbreviations: CAL, cerebellum anterior lobe; CN, cerebellum network; DMN, default mode network; ECN, executive control network; L, left; MFG, middle frontal gyrus; MNI: Montreal Neurologic Institute; R, right; SFG, superior frontal gyrus; SN, salience network; SPL, superior parietal lobe; VN, visual network.

### 3.4 Group differences in static FNC

**Figure 3:**
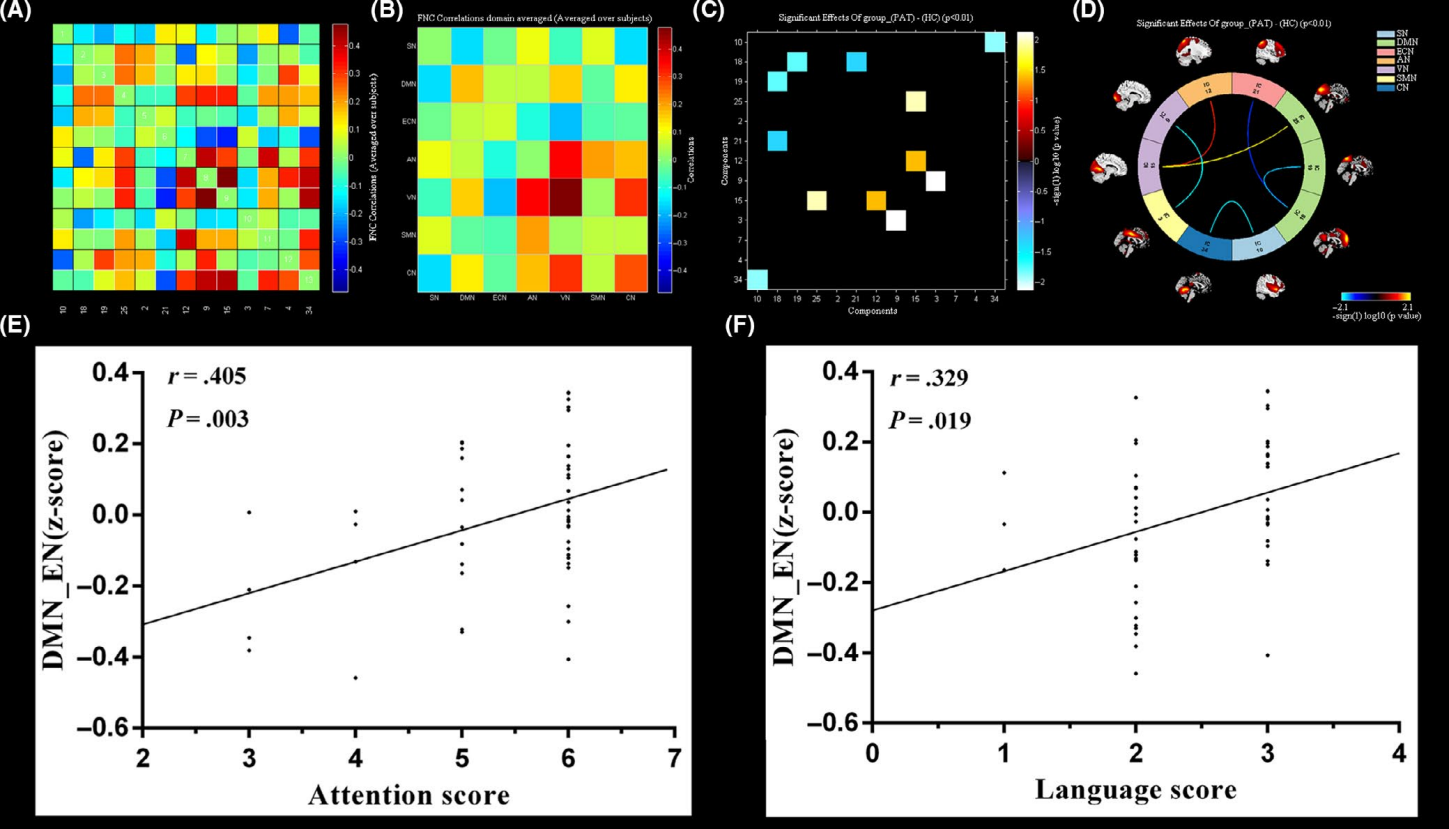
Group differences in static FNC. (A) FNC correlations matrix (averaged over subjects); (B) FNC correlations domain matrix (averaged over subjects); (C) significant effects of group between patients and controls; (D)five connections were found to be significantly altered (*P* < .05); and DMN‐ECN connection was found to be positively correlated with attention scores (*r* = .405, *P* = .003) (E) and language scores (*r* = .329, *P* = .019) (F)

For the FNC analysis, five connections were found to be significantly altered. Relative to the HC group, the mTBI group exhibited significantly decreased negative interactions in three RSN connections, including the SN‐CN connection, VN‐SMN connection, and ECN‐DMN connection. Moreover, compared with the HC group, the mTBI group showed increased FNC in the interaction between the VN cortices (IC15) and AN cortices (IC12), as well as the VN cortices (IC15) and DMN cortices (IC25). In addition, DMN (IC18)‐DMN (IC19), that is, FC within an RSN, was also found to be significantly decreased in the mTBI group compared with the HC group (Figure 3A‐D).

## 4 CORRELATION ANALYSIS

**Figure 4:**
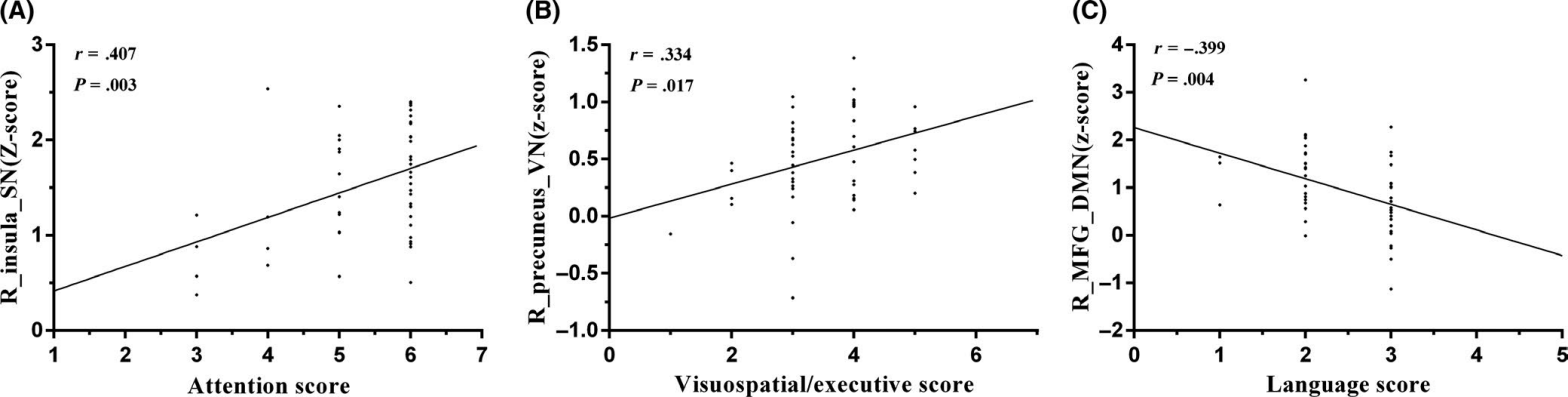
Correlations between the mean z‐scores of six ROIs in the five RSNs and cognitive assessment scores in mTBI patients at the acute stage. (A) Significant positive correlations were found between the right insula region within the SN and attention scores (*r* = .407, *P* = .003); (B) significant positive correlations were found between the right precuneus within VN and visuospatial/executive scores (*r* = .334, *P* = .017); and (C) significant negative correlation was found between the right MFG within the DMN and language scores (*r* = −.399, *P* = .004)

Correlations were performed between the mean z‐scores of six ROIs in the five RSNs and cognitive assessment scores, and significant positive correlations were found between the right insula region within the SN and attention scores (*r* = .407, *P* = .003), as well as the right precuneus within VN and visuospatial/executive scores (*r* = .334, *P* = .017). In addition, a significant negative correlation was found between the right MFG within the DMN and language scores (*r* = −.399, *P* = .004) (Figure 4). Moreover, after performing the correlations between the FNC coefficients (five connections) and the cognitive assessment scores in the mTBI group, only the DMN‐ECN connection was found to be positively correlated with attention scores (*r* = .405, *P* = .003) and language scores (*r* = .329, *P* = .019) (Figure 3E‐F).

## 5 DISCUSSION

In this study, decreased FC was observed within several motor‐ and cognitive‐related networks in acute mTBI, that is, the SN, ECN, VN, and CN. It is worth pointing out that the change in the SN in acute mTBI was an important finding that had not been previously reported in acute mTBI. The SN is a large‐scale edge network that coactivates signals needed for behavioral changes.^27^ As part of the SN, the insular cortex plays an important role in integrating external and internal processes, emotional processing, and controlling cognitive functions and behavior.^28^ Our group recently demonstrated FC dysfunction in the insula of mTBI patients.^29^, ^30^ In addition, the frontoparietal lobe is an important network of spatial attention, and the ECN is considered to have a good correspondence with the cognitive language paradigm.^21^ Consistent with this notion, mTBI patients showed decreased FC in the right insula within the SN as well as the left SPL and the left SFG within the ECN, suggesting that dysfunction within the SN and ECN might contribute to the cognitive impairments observed in acute mTBI patients.

In general, the VN is associated with the processing of visual information, and visual dysfunction in mTBI patients has been associated with high levels of processing deficits.^31^ Moreover, the positive correlation between the VN and visuospatial/executive scores also suggested that decreased FC in the VN aggravated the cognitive dysfunction in acute mTBI. In addition, an understanding of the cerebellum's role in motor learning and cognitive processes has been emerging, and cerebellar damage might lead to postural and movement impairments.^32^ Previous studies have demonstrated that mTBI patients in the acute stage had abnormal fractional anisotropy in specific regions of the cerebellum, and altered cerebellar fractional anisotropy was associated with cognitive impairment.^33^ Within the CN, acute mTBI patients showed decreased connectivity in the right CAL in this study. This finding was not consistent with the study of Wang et al, suggesting that acute mTBI patients showed increased FC in the CN.^10^

A fMRI study demonstrated hyperconnectivity within DMN connectivity in mTBI patients,^5^ which was mainly located in portions of the posterior cingulate and medial prefrontal cortex, representing brain neuroplasticity operative in neural repair and recovery after injury. In line with these reports, we found that the left MFG appeared hyperconnected within the DMN in the mTBI patients in the acute stage, and this result could be interpreted as a compensatory reallocation or recruitment of cognitive resources. Meanwhile, our previous study used GCA to analyze the causal connections in the acute mTBI patients and demonstrated significantly increased connectivity from the left ACC to the left MFG.^34^ For mTBI patients, Benier et al and Sharp et al hypothesized that such hyperconnectivity patterns in the DMN could be related to dysfunction in working memory and attention switching during cognitive demand.^35^, ^36^ MFG connectivity appeared to be associated with the degree of language deficit, a finding supported by the notion of increased use of MFG neural resources as a compensation for impaired neural cognitive function.

The SN has been described as a task‐positive network that activates corresponding areas during cognitive task performance, while the cerebellum is the center of motor planning and control. Previous study has found that changes in network interactions between the DMN and SN were associated with cognitive impairment after mTBI.^37^ The reduced FC among the DMN, ECN, and SN suggested that there was an inefficient balance and regulation among these networks, which may imply that network inhibition or network imbalance is a possible mechanism for acute cognitive impairment in mTBI. In addition to FNC abnormalities, we also found a negative correlation between the DMN‐ECN connection and attention scores, as well as language scores, suggesting that the uncoupling between the DMN and ECN might relate to the disrupted cognitive self‐regulation in acute mTBI.

Many parts of visual cortex are located in areas vulnerable to mTBI, and almost 70% of sensory processing in the brain is related to vision.^38^ Visual perception dysfunction reflects impaired visual information processing ability, which occurs frequently in mTBI.^39^ Since the frontoparietal network is pivotal in visual‐spatial attention and motor function, the disconnection of the VN‐SMN, VN‐AN, and VN‐DMN pathways might be one factor of visual perception impairments in acute mTBI patients. Therefore, we believe that the changes in the interactions between these important networks may provide further information on how patients can adjust the connectivity of the related networks during cognitive tests.

Our study has several limitations. First, this is a preliminary cross‐sectional study of FC changes in acute mTBI with limited sample size so that it is difficult to directly infer the causal relationship between brain functional network and cognitive impairment in mTBI. More longitudinal studies with larger samples are needed. Second, our study was based on a limited network interaction model. There are other networks that have not been considered yet may play an equally important role in the pathophysiology of cognitive impairment in acute mTBI, such as the auditory network (AuN) and the self‐referential network (SRN). Moreover, although we have excluded the mTBI patients and HCs who complain about visual impairment, the visual acuity/corrected visual acuity was not measured. The visual function between groups will be assessed in our future research. Finally, despite the advantages of using ICA to reveal unconstrained brain connections, ICA cannot reveal the directionality of the interactions between networks. Further studies are required to assess the direct impact and direction of each network in coupling with other networks in mTBI.

In conclusion, this study demonstrated widespread early changes in intra‐ and inter‐static FNC in the acute stage after mTBI. These network interactions can provide a powerful way of evaluating and predicting cognitive impairment after mTBI and contribute to our understanding of the neural mechanisms of cognitive impairment after mTBI.

## CONFLICT OF INTEREST

The authors declare no conflict of interest.

## ETHICAL APPROVAL

The current study was approved by the Institutional Review Board of Nanjing Medical University. Written informed consent was obtained from all participants before their participation in the study protocol.
